# UV radiation at 222, 254, and 282 nm inhibits sporulation and suppresses infectivity of *Eimeria acervulina* oocysts

**DOI:** 10.1128/spectrum.02439-24

**Published:** 2025-02-06

**Authors:** Aaron A. Baumann, Addison K. Myers, Niloofar Khajeh-Kazerooni, Ashley Wise-Mitchell, Benjamin M. Rosenthal, Mark C. Jenkins, Celia O'Brien, Lorraine Fuller, Samuel Tremaine, Mark Morgan, Scott C. Lenaghan

**Affiliations:** 1 Center for Agricultural Synthetic Biology (CASB), University of Tennessee, Knoxville, Tennessee, USA; 2 Department of Food Science, University of Tennessee, Knoxville, Tennessee, USA; 3 Animal Parasitic Disease Laboratory, Agricultural Research Service, US Department of Agriculture, Beltsville, Maryland, USA; 4 Department of Poultry Science, University of Georgia, Athens, Georgia, USA; 5 Herbert College of Agriculture, Department of Plant Sciences, University of Tennessee, Knoxville, Tennessee, USA; University of Minnesota Twin Cities, St. Paul, Minnesota, USA

**Keywords:** Coccidia, UV radiation, *Eimeria*, *Cyclospora*, poultry, sporulation

## Abstract

*Eimeria acervulina* is one of several apicomplexan parasites that can cause the economically important disease avian coccidiosis, which is transmitted *via* the fecal-oral route. It is also a valuable model for exploring interventions capable of reducing the public health risks posed by the related foodborne parasite *Cyclospora cayetanensis*. Unsporulated (noninfectious) oocysts are shed in the feces and sporulate in the environment to become infectious. In this study, the effect of UV radiation (UVR) on *E. acervulina* sporulation efficiency was evaluated. Nonstandard wavelengths including 222 and 282 nm have been established as alternatives to standard 254 nm UVR for sterilization. To address the disinfection capability of these nonstandard wavelengths, *E. acervulina* sporulation was scored following treatment under three wavelengths: 222, 254, or 282 nm. Each wavelength significantly (*P* < 0.05) reduced the sporulation rate relative to untreated controls, supporting the utility of nonstandard UVR treatments for coccidian inactivation. Furthermore, infectivity assays showed that chickens administered *E. acervulina* treated at each wavelength shed significantly fewer oocysts (*P* < 0.05) than those that received untreated oocysts, demonstrating a near 3-log reduction and verifying that these alternative wavelengths successfully break the transmission cycle. Owing to its biological similarity with the emerging enteric parasite *Cyclospora cayetanensis*, it is proposed that disinfection with 222 or 282 nm UVR will likewise be efficacious against *Cyclospora*.

**IMPORTANCE:**

Coccidian parasites cause enteric disease in animals and people. For example, *Eimeria acervulina* imposes major economic burdens on the poultry industry and provides a surrogate for investigating means to mitigate the foodborne risk that *Cyclospora cayetanensis* poses to human health. Previous work established that UV radiation at 254 nm can inactivate the oocysts of *E. acervulina,* but radiation at this wavelength harms human skin and eyes. Since nonstandard UVR at wavelengths of 222 and 282 nm shows promise against pathogens like *Giardia* and *Cryptosporidium,* the present work sought to determine whether such exposures could arrest *E. acervulina* development and reduce their infectivity. These nonstandard wavelengths proved capable of disrupting transmission. Epidemiological evidence suggests *Cyclospora* transmission through the food chain; the use of nonstandard UV wavelengths represents a promising method to inactivate coccidian oocysts, thereby protecting produce supply chains while, specifically in the case of 222 nm, incurring less risk to occupational health.

## INTRODUCTION

Coccidiosis is a major, economically important poultry disease. In 2022, the Association of Veterinarians in Broiler Production listed avian coccidiosis as the most important disease in poultry (1). Characterized by enteritis, diarrhea, dehydration, and weight loss, this disease causes an estimated $12 billion annual loss globally (2). Coccidiosis is caused by members of the apicomplexan genus *Eimeria* including *E. acervulina*, *E. tenella*, *E. maxima,* and others (3). Among these, *E. acervulina* colonizes the small intestine causing malabsorptive coccidiosis that leads to stifled weight gain and inefficient feed conversion (4). *E. acervulina* has a complex life history characterized by transmissible, infective (sporulated), and metabolically inert (unsporulated), noninfective stages. The environmentally stable, metabolically inert, unsporulated oocyst is shed in the feces and sporulates in the environment, during which the infectious sporozoites develop. This transition is accompanied by a drastic change in morphology whereby the cytoplasmic mass reorganizes and four clearly defined, ovoid sporocysts are formed. Since only sporulated oocysts are infectious, disinfection methods that block sporulation represent attractive strategies to control transmission.

Like *E. acervulina*, the emerging human enteric pathogen *Cyclospora* is transmitted *via* the fecal-oral route and must sporulate to achieve infectivity (5). Cyclosporiasis is a severe diarrheal illness characterized by dehydration, suppressed appetite, nausea, fatigue, and weight loss (6) and these symptoms can be especially severe in children or immunocompromised individuals (7). Recurring multistate cyclosporiasis outbreaks (8) highlight the urgent need for enhanced diagnostic and control methods. However, many aspects of *Cyclospora* biology remain unresolved due to several constraints. First, oocysts are shed intermittently, and case reporting is often delayed due to the extended incubation period (median 7.5 days [9]). In a clinical setting, cyclosporiasis may be overlooked in patients presenting with diarrheal disease; by the time viral or bacterial agents are eliminated as culprits, oocysts may no longer be shed. Therefore, the parasites are challenging to obtain in sufficient quantity for study. Second, samples obtained through public health departments are often treated with fixatives and cannot be used for gene expression studies or inactivation trials. Third, *Cyclospora* cannot be cultured *in vitro* or in any non-human host (10). A trial involving willing human volunteers failed to yield viable oocysts (11), compounding the challenges in obtaining viable *Cyclospora* oocysts.

In efforts to untangle *Cyclospora* biology, researchers have adopted *E. acervulina* as a surrogate, owing to close phylogenetic relationships and considerable biological similarities. Moreover, *E. acervulina* poses no human health risks and can be propagated in great quantity (12, 13). It is expected that disinfection protocols showing efficacy in the surrogate will also find utility for *Cyclospora* inactivation. Previous studies have shown that *Cyclospora* oocysts are sensitive to thermal treatment (14) and corrosive chemical agents like hypochlorite ions. However, *Cyclospora* transmission has been epidemiologically linked to the produce supply chain, sanitation methods must retain the quality of the produce vehicle. UV radiation (UVR) is effective against a wide range of microorganisms including viruses, bacteria, and protozoa and unlike thermal treatments, UVR does not compromise the sensory aspects of production. In fact, UVR exposure can provide benefits including increased antioxidant capacity (15).

The UV spectrum is subdivided into UVA (320–400 nm), UVB (280–320 nm), and UVC (200–280 nm) ranges. 254 nm UVC is widely used as a germicidal agent, and its neutralizing activity against protozoa is well documented (16–18). Using viability dyes and flow cytometry, Adeyamo et al. (19) showed that *Cryptosporidium* is more efficiently inactivated by UVR than by chlorine gas. In another study, raspberry and basil samples spiked with UVR-treated *E. acervulina* were fed to birds, who remained asymptomatic for coccidiosis and shed fewer oocysts than those challenged with nontreated oocysts (20). El Ashram et al. (21) demonstrated that oral administration of *Eimeria* treated with 254 nm UVC for 1 hour significantly decreased oocyst shedding, reduced lesions attributed to coccidiosis, and increased body weight relative to birds receiving untreated oocysts. Similarly, Djemai and colleagues (22) used 254 nm UVR inactivated *Eimeria* oocysts to immunize birds against coccidiosis.

Despite its utility, germicidal 254 nm UVR is harmful to nontarget organisms. This reality has promoted the exploration of alternative wavelengths in the UV spectrum to find an appropriate tradeoff between disinfection activity and nontarget exposure risk. Radiation in the far-UVC range cannot penetrate the outer, non-living layer of the skin or eye (23) and poses much less risk to nontarget organisms including humans and livestock. By contrast, by virtue of their size, bacteria, protozoa, and viruses are susceptible to physiological damage from far UVC exposure. 222 nm UVR effectively killed methicillin-resistant *Staphylococcus aureus* (MRSA), and tests on both a 3D human skin model (EpiDerm [24]) and mouse epidermis supported reduced exposure risks (25). In a model of surgical site infection, 222 nm was used to treat MRSA-infected mice, significantly reducing bacterial load. But unlike 254 nm UVR, 222 nm UVR did not hinder the keratinocyte movement crucial for wound healing (26). Owing to its relative safety, UV sources emitting at 207 or 222 nm have been deployed in open spaces to effectively inactivate aerosolized bacteria (27) and enveloped viruses (28). In addition to 222 nm, the disinfection potential of UVR at 282 nm has also been explored. In contrast to a body of evidence supporting the relative safety of 222 nm UVR exposure, little work has been done to extensively quantify the health risks associated with exposure to 282 nm UVR when used as a disinfecting agent. Nevertheless, devices outputting 282 nm UVR have been employed in occupied public spaces. Both 222 and 282 nm UVR were used to inactivate *E. coli* and *E. faecalis* bacteria in water, and while both wavelengths demonstrated germicidal activity, 222 nm was consistently more effective than 282 nm (29). Both 270 nm and 222 nm UVR outperformed 254 nm UVR for inactivating biofilm-bound *Pseudomonas aeruginosa*, indicating that the absorptive properties of the substrate can influence the activity series of the wavelengths (30).

While alternative UV wavelengths have been comprehensively evaluated for the disinfection of bacteria and viruses, relatively few studies have evaluated their utility against coccidians. Among these methods, an infectivity assay revealed that polychromatic UV light, emitting across various wavelengths from 248 to 295 nm, was an effective means for inactivating *Cryptosporidium* oocysts. The authors concluded, with 95% confidence, that UV doses of 7.5 and 11 mJ cm^−2^ are sufficient to deactivate *Cryptosporidium* to an average level of 90% and 99%, respectively (31). Interestingly, Takahashi and colleagues (32) showed that *Cryptosporidium* shed patterns in an immunodeficient mouse model were significantly delayed when the oocysts were treated with LEDs emitting at 284 and 289 nm vs. the shed patterns obtained following treatment with a conventional mercury lamp emitting at 254 nm. Furthermore, excystation assays, coupled with viability dyes, showed that UV LEDs emitting at peak wavelengths of 268, 275, 284, and 289 nm inactivated *Cryptosporidium* after exposure for as little as 30–40 minutes, with 298 nm UVR showing the greatest activity (33). Since protein absorbs at 280 nm, the authors suggest that the longer wavelengths may disrupt the protein fraction of the sporocyst wall and hinder sporozoite release. Indeed, a biochemical analysis of coccidian oocyst and sporocyst walls showed these structures are composed of greater than 90% protein (34).

Here, *E. acervulina* was employed as a surrogate for *Cyclospora* (13, 20) to investigate the impact on oocyst sporulation following UVR exposure at 222, 254, and 282 nm. Since *Cyclospora* currently lacks an infectivity model, and since sporulation is necessary for infectivity (5), this metric remains the only suitable method for evaluating *Cyclospora* inactivation. Also, since *E. acervulina* can be propagated *in vivo*, an infectivity assay was performed to validate a reduction in infectious potential concomitant with suppressed sporulation efficiency. The findings presented in this study suggest promise for nonstandard UVR treatment of contaminated surfaces to mitigate the risk of *Cyclospora* transmission.

## RESULTS

### Sporulation inhibition

**Fig 1:**
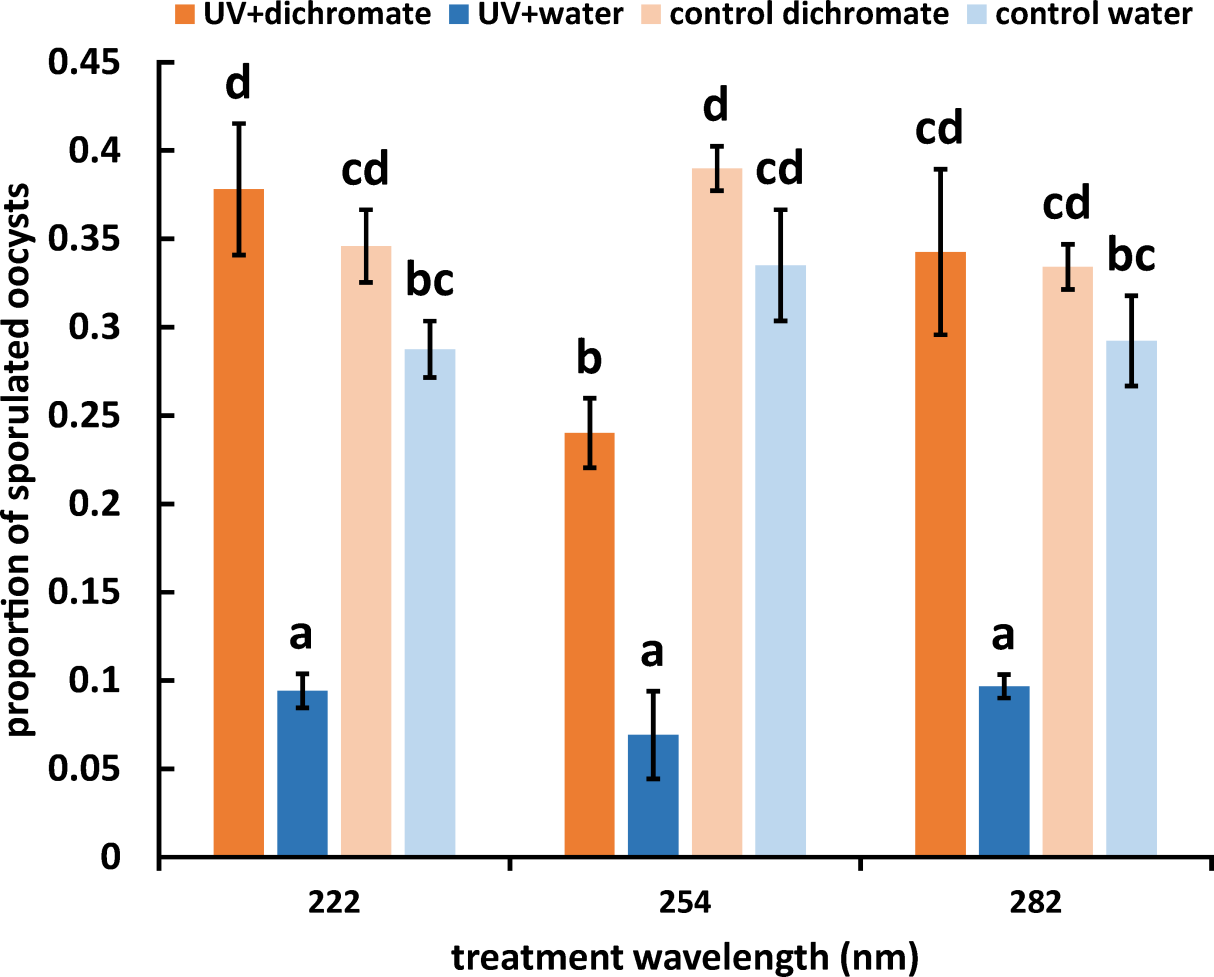
Irradiation at all three UV wavelengths suppresses sporulation in parasites suspended in water lacking potassium dichromate. The proportion of sporulated to total oocysts was scored across four treatments: UVR applied to oocysts in 2.5% wt/vol potassium dichromate suspension, UVR applied to oocysts suspended in water, no UV control oocysts suspended in 2.5% wt/vol potassium dichromate, and no UV control oocysts suspended in water. Three wavelengths, 222, 254, and 282 nm were used for 5 minutes each at an irradiance of 20*10^−4^ mW cm^−2^. Oocysts were treated in 100 μL (1 × 10^5^ oocysts/mL) volumes, representing 50 μL of oocyst suspension mixed with 50 μL of dichromate or water. Bars represent mean ± SEM. Letters above bars represent statistical significance at *P* < 0.05, ANOVA with Waller-Duncan post hoc adjustment.

**Fig 2:**
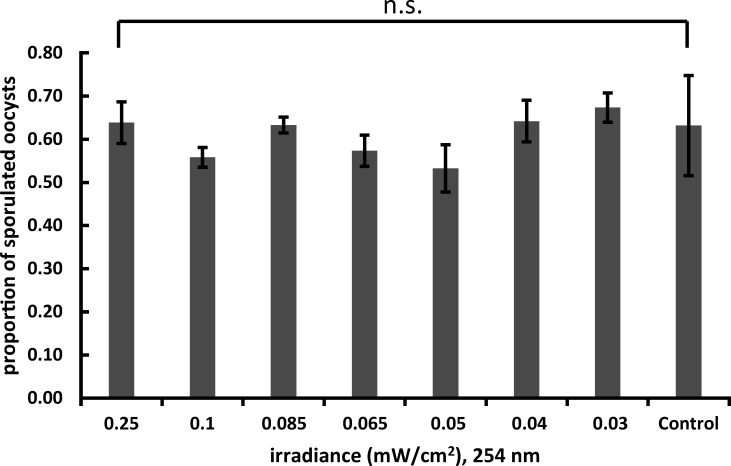
Shielding oocysts with a polystyrene lid prevents UV irradiation from arresting sporulation. Proportion of sporulated oocysts after a 1 minute treatment under 254 nm in well plates with transparent polystyrene lids. Bars: mean ± SEM. n.s. = not significant by ANOVA.

An initial 5-minute screening, to determine the efficacy of 254 nm vs. alternate wavelengths, showed that 254 nm, 222 nm, and 282 nm UVR all achieved statistically similar inhibition activity against sporulation in *E. acervulina* (relative to untreated controls), resulting in an approximately 1-log reduction (Fig. 1). Irradiating oocysts in water affected strong inhibition of sporulation; potassium dichromate, which is used in standard protocols for sporulating coccidian oocysts, provided protective effects against UVR-induced inhibition (Fig. 1). In the presence of 2.5% potassium dichromate, treatments with 222 and 282 nm sporulated at rates comparable to those not exposed to UV irradiation, toward a 40% sporulation efficiency. Exposure to 254 nm irradiation reduced sporulation rates even in those protected by potassium dichromate, to approximately 25%, but greater reductions in sporulation resulted when potassium dichromate was absent. In the absence of UVR exposure, the presence of potassium dichromate only modestly increased sporulation rates (Fig. 1). In contrast to oocysts treated in open air, oocysts that were treated in well plates equipped with transparent polystyrene lids (protected from exposure) sporulated at rates statistically equivalent to untreated controls, demonstrating the necessity for direct UVR exposure to elicit developmental arrest (Fig. 2). The turbidity of each suspension as measured by taking an OD_600_ was as follows (average and standard deviation of three independent readings): oocyst suspension (1.795 ± 0.07); oocyst: dichromate 1:1 vol:vol (1.665 ± 0.113); dichromate alone (0.070 ± 0.01); oocyst:water 1:1 vol:vol (1.377 ± 0.05); water (0.034 ± 0.003).

**Fig 3:**
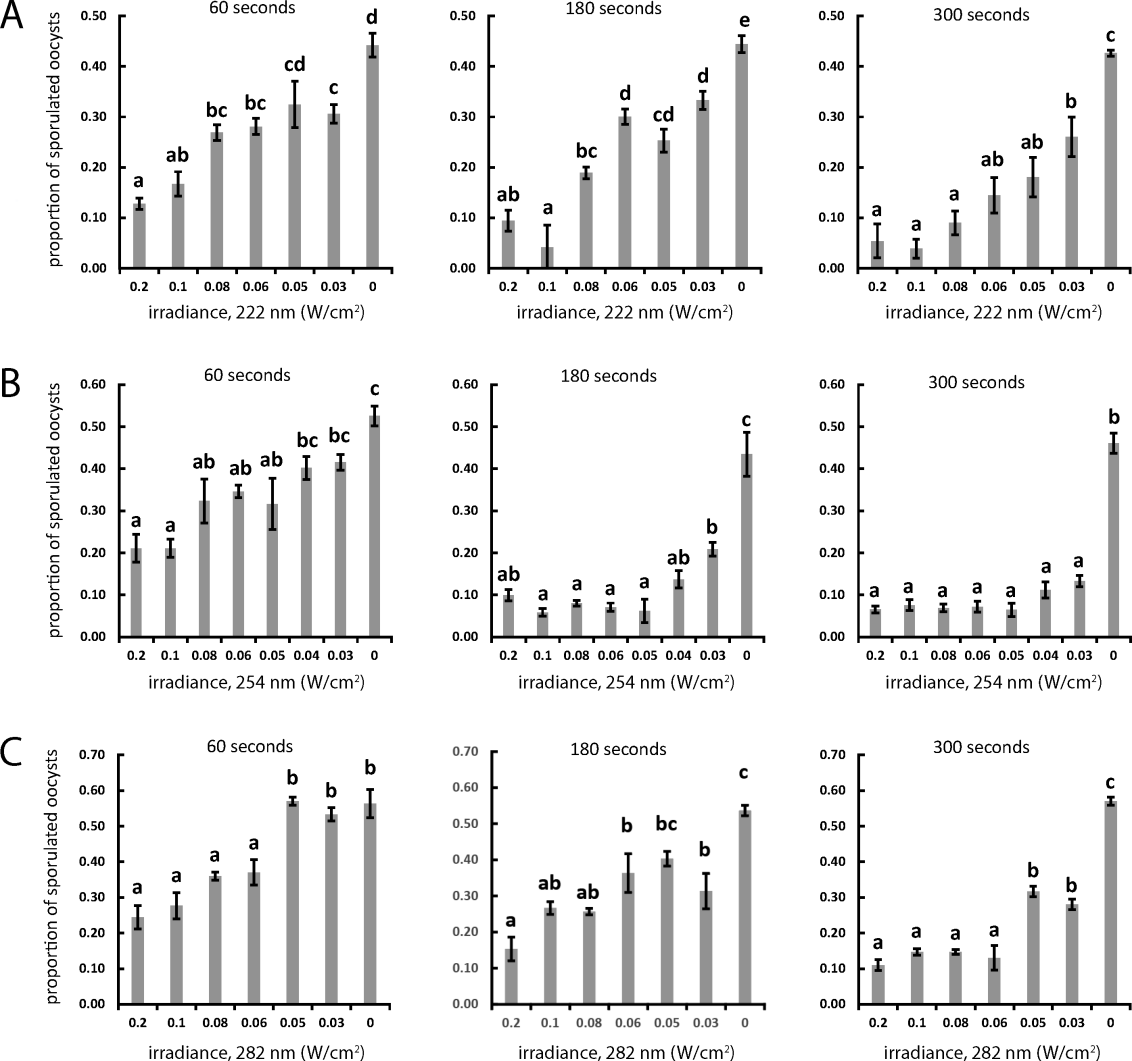
Sporulation responses to UV irradiation at three UV wavelengths as distance and duration of exposure varies. The proportion of sporulated oocysts per sample relative to irradiance under 222 nm (**A**), 254 nm (**B**), or 282 nm (**C**) at several distances calibrated to provide the indicated irradiance (X-axis) for 60, 180, or 300 seconds. Bars: mean ± SEM. Letters represent statistical significance at *P* < 0.05, one-way ANOVA with Tukey post hoc correction.

**Fig 4:**
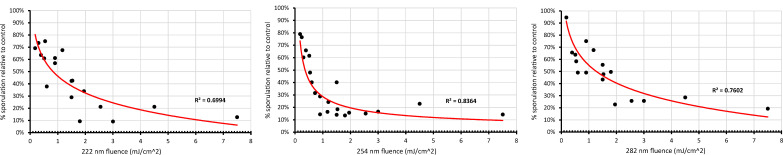
% sporulation relative to non-treated controls as a function of fluence for UVR at 222 (left), 254 (center), and 282 nm (right). To predict approximate LC_50_ values, best-fit curves were generated using Microsoft Excel.

Next, oocysts were exposed to UVR at each wavelength for various time intervals and at various distances to establish dosage gradients under three contact times. 222, 254, and 282 nm UVR all significantly reduced the sporulation capacity of *E. acervulina* oocysts in a dose-dependent manner (Fig. 3). In the 254 nm trial, after subtracting the baseline sporulation rate, or the sporulation rate recorded after oocyst purification, the average sporulation rate in untreated controls was observed as 47.4% (range: 43.5%–52.5%, *n* = 3 groups of >500 oocysts). The minimal dose of 254 nm UVR that was required to significantly inhibit sporulation was approximately 3 mJ cm^−2^, corresponding to treatment for 60 seconds under an irradiance of 0.05 mW cm^−2^ (Fig. 3B). Under 222 and 282 nm UVR, following baseline subtraction, the average sporulation rates in untreated controls were recorded as 47.3% (range: 42.6%–44.4%) and 55.7% (range: 53.7%–57%), respectively. At 222 nm, the minimal dose required for significant sporulation inhibition relative to controls was 1.8 mJ cm^−2^ (0.03 mW cm^−2^ for 60 seconds) (Fig. 3A and C), but the next highest treatment was statistically similar to controls, showing some variability in either oocyst response or bulb performance (Fig. 3A, left panel). The minimal dose for significant reduction of sporulation at 282 nm was 3 mJ cm^−2^, corresponding to treatment with 0.05 mW cm^−2^ for 60 seconds (Fig. 3B, left panel). For all three wavelengths, the maximum offset distance between the sample and UV source, chosen according to measurements *via* the ILT-1700 radiometer to correspond to an irradiance of 0.03 mW cm^−2^, was sufficient to significantly reduce sporulation relative to controls after treatment for 180 seconds. Overall, treatment at 180 or 300 seconds under 254 nm (Fig. 3B) showed higher efficacy in blocking sporulation than the same treatments under 222 nm or 282 nm UVR. For each distance and time combination, the radiant energy in mJ cm^−2^ was calculated and this value was plotted against the percentage of sporulated oocysts at each value (Fig. 4). In each case, the approximate radiant energy required to achieve 50% inhibition of sporulation relative to controls was between 0.5 and 1.0 mJ cm^−2^.

### Infectivity assay

Sporulation does not always indicate infectious potential. Therefore, an *in vivo* infectivity assay was employed to evaluate whether UVR-treated oocysts showed similar infectious potential to untreated oocysts. Chickens were inoculated either with oocysts treated at one of two UVR doses under each of the three wavelengths for 300 seconds or with oocysts that received no UVR exposure. Chickens inoculated with oocysts treated under each of the three wavelengths showed significantly lower oocyst shedding than chickens inoculated with untreated oocysts (*P* < 0.05). The mean oocyst count from chickens that received untreated oocysts (*n* = 3) was 207.5 × 10^5^ oocysts (Table 1). Chickens fed oocysts undergoing the least effective UV treatment (222 nm, 19.5 mJ cm^−2^) yielded 36.1 × 10^5^ oocysts, an 82% or 0.76-log reduction of over 17 million oocysts. The treatment most effective in reducing oocyst production by chickens (282 nm at 0.065 mW cm^−2^ for 300 seconds, for a fluence of 19.5 mJ cm^−2^) yielded only 0.7 × 10^5^ oocysts, a reduction of close to 3-log at over 20 million, or >99.6%. Therefore, UVR at 222, 254, and 282 nm markedly suppresses the infectivity of *E. acervulina* oocysts.

**TABLE 1:** Average *Eimeria acervulina* oocyst output from chickens (*n* = 3/treatment) inoculated with 500 oocysts exposed to three different UV wavelengths (222, 254, or 282 nm) and two different irradiances (column 2) for 300 seconds^*a*^

Group #	Treatment fluence rate (mW cm^−2^)	*Eimeria acervulina* oocysts output (mean + S.D.) × 10^5^	*Eimeria acervulina* oocysts output (CV)
1	222–0.065	36.1 ± 21.4	0.6
2	222–0.125	1.4 ± 0.4	0.3
3	254–0.065	9.0 ± 3.5	0.4
4	254–0.125	1.2 ± 0.6	0.5
5	282–0.065	0.7 ± 0.5	0.8
6	282–0.125	3.2 ± 4.1	1.3
7	No UV	207.5 ± 125.3	0.6

^*a*^ Significant difference by ANOVA (*P* < 0.01) between irradiated (groups 1–6) and control (group 7) oocysts, ranging from >82% to >99% mean reductions. No significant difference (*P* > 0.05) was observed among groups 2–6, whose minimum reduction was >95%. The far-right column shows the coefficient of variation (S.D./mean); CV, coefficient of variation

## DISCUSSION

In this study, purified *E. acervulina* oocysts were exposed to a range of UV wavelength, and the effects on sporulation rate and infectious potential were measured. Far-UV spectrum LEDs have been investigated thoroughly for their utility in combating the spread of viral infectious disease in recent years due to the SARS-Cov-2 pandemic. A general trend has suggested that shorter wavelength UVC radiation is more effective than longer wavelength treatments (e.g., <280 nm) for inactivating SARS-CoV-2 (35, 36) and bacteria (29). This study demonstrated that UVR at wavelengths 222, 254, and 282 nm can each similarly suppress the infectivity of the poultry parasite *E. acervulina*. Owing to its considerable biological similarity to *C. cayetanensis*, nonconventional UVR wavelengths would likely also inactivate *Cyclospora* oocysts. The sporulation rate in *E. acervulina* was suppressed in a manner that was dependent on both the distance from and the time under the UVR source. 254 nm UVR showed the best activity against sporulation under the times and distances tested, relative to 222 or 282 nm. Notably, a higher dose of 282 nm UVR led to higher oocyst output compared to the lower dose in the infectivity assay (Table 1). Given the clear trends in the inactivation trials showing higher doses lead to lower rates of sporulation (and presumably infectivity), this result likely reflects the inherent variability in *Eimeria*’s response to UV. Figure 4A and B likewise shows instances in which the sporulation efficiency was higher in one instance under a high dose than under a low dose of UVR, despite a strong trend supporting a dose- and time-dependent effect of UVR in promoting developmental arrest.

The mechanism of UVR-induced cellular damage has been studied extensively in mammalian systems, particularly with respect to the solar range of the UV spectrum; UVC (200–280 nm) is blocked by ozone in the stratosphere. Human skin is a multi-layered structure that harbors several lines of defense against UVR, including the stratum corneum that blocks 70% of UVB penetration, following which DNA repair enzymes, pro-apoptotic proteins, and cellular immunity are employed (37). UVA exposure from 320 to 340 nm (UVA2 range) produces reactive oxygen species and can inhibit DNA repair enzymes (38), and both UVA and UVB contribute to the production of wavelength-specific bipyrimidine photoproducts (39). If not repaired, these dimers induce the pro-apoptotic protein p53, leading to cell death (40). However, the mechanism of UV inactivation of coccidian oocysts is unclear. Some inactivation methods, like freeze-thaw cycling or treatment with essential oils (41), can visibly damage the oocyst wall, resulting in the expulsion of cytoplasmic contents. McGuigan et al. (42) report physical disruption of the *Cryptosporidium* wall after solar exposure for at least 10 hours with a calculated optical dose of approximately 30 kJ. In this study, which employed substantially lower doses of UVR, morphological examination by light microscopy revealed no apparent aberrations in oocyst wall integrity. However, electron microscopy was not employed. It is likely, given the deep evolutionary conservation of UV response mechanisms, that similar molecular actors are involved in UV response in *E. acervulina*. Studies focusing on rad-family members and proteins responsible for DNA repair may unravel the molecular response to UV in protozoa.

The primary challenge to achieving disinfection using UV radiation lies in effectively penetrating barriers to contact. These barriers include the oocyst and sporocyst walls and any physical barrier isolating the target organism from UVR. Successful inactivation of oocysts in this study was achieved by treating them in a suspension of water with no physical barriers separating the parasites from the source of UV emission. This study has established that UV at 222, 254, and 282 nm is effective both in blocking sporulation and reducing infective potential but fails to account for the reality that *E. acervulina* and *Cyclospora* may reside within complex matrices with impenetrable surfaces or layers. This limitation was clearly demonstrated when oocysts treated under 254 nm in well plates with polystyrene lids showed no significant reduction in sporulation (Fig. 2). Since *E. acervulina* is shed in chicken feces, oocysts may be shielded from the dose of penetrating radiation required to achieve sporulation inhibition or cell death, thus limiting the utility for UVR in a broiler house. *Cyclospora* may likewise be sequestered from UV exposure within the complex surface of a produce matrix, for instance between individual drupelets of a raspberry. These effects might be mitigated by decreasing the distance between the UV light and the matrix, or incorporating a light-scattering ballast so that incident light is reflected across surfaces more evenly.

Recently, it was shown that aqueous ozone also inhibits *E. acervulina* sporulation in a dose-dependent manner (43). There is an obvious synergism between these methods that could lend to reduced contact time on produce, processing equipment, by using ozonated water to wash potentially contaminated material under a far UVC emitting LED matrix. Using ozone and UV alone or in combination, Qiao and colleagues (44) report that the combined use of gaseous ozone and UV led to a significant decrease in the required disinfection time versus either method alone and found that this combination effectively eliminated the inhibitory effect of barriers to UV contact. Such a combinatorial approach could find utility in treating packing materials, washing surfaces, or other sources of contact contamination. Furthermore, both ozone and UVR have demonstrated sensory and quality benefits when used to treat fresh produce. 222 nm UVR reduced the content of the mycotoxin patulin in apple juice by 90%, with no significant changes to pH, soluble solids, or juice color (45), and lotus root treated at 254 nm showed a reduction in browning over time and this was achieved through suppression of polyphenol oxidase and peroxidase activity (46).

### Conclusion

UVR is an effective means for controlling a variety of microorganisms. Importantly, UVR at nonstandard wavelengths presents far fewer exposure risks than the conventional 254 nm wavelength. Furthermore, its use for food sanitation is warranted since UVR subverts the necessity for harsh thermal or chemical treatments that could compromise produce integrity. The findings herein indicate that UVR at nonstandard wavelengths both suppresses the sporulation and infectivity of *E. acervulina*. Owing to their close phylogenetic relationship, nonstandard far UV wavelengths will likely find utility in combatting the spread of domestically and internationally acquired cases of cyclosporiasis.

## MATERIALS AND METHODS

### Oocyst preparation and UV treatments

HR308 broiler chicks (Longeneckers Hatchery, Elizabethtown, PA) were inoculated with *Eimeria acervulina* oocysts following standard protocols (13). Chicken feces containing oocysts were mixed with tap water at a ratio of 1:5 and stirred for 30 minutes using a magnetic stir bar. The mixture was passed through cheesecloth placed in a funnel, collected in a large beaker, and aliquots were provisioned into 50 mL conical tubes. The suspension was centrifuged at 935 × *g* for 10 minutes using a Thermo Scientific TX-1000 rotor (Thermo Scientific X Pro Series, r = 20.9 cm). Pellets were resuspended in saturated NaCl solution (360 g/L), centrifuged again at 935 × *g* for 10 minutes, and allowed to rest on the benchtop for 10 minutes. Top fractions of approximately 1 mL were collected, washed with tap water, and centrifuged at 1460 × *g* for 15 minutes. This wash step was performed three times to remove residual salt. Finally, pellets were combined and resuspended in 50 mL of tap water. 50 µL of the purified oocysts was added to each well of a 96-well plate and exposed to either 222, 254, or 282 nm UVR at a concentration of 1 × 10^5^ /mL (1:10 dilution of the original purified concentration) in either water or potassium dichromate as described below. Untreated controls were shielded from UVR but exposed to ambient light.

**Fig 5:**
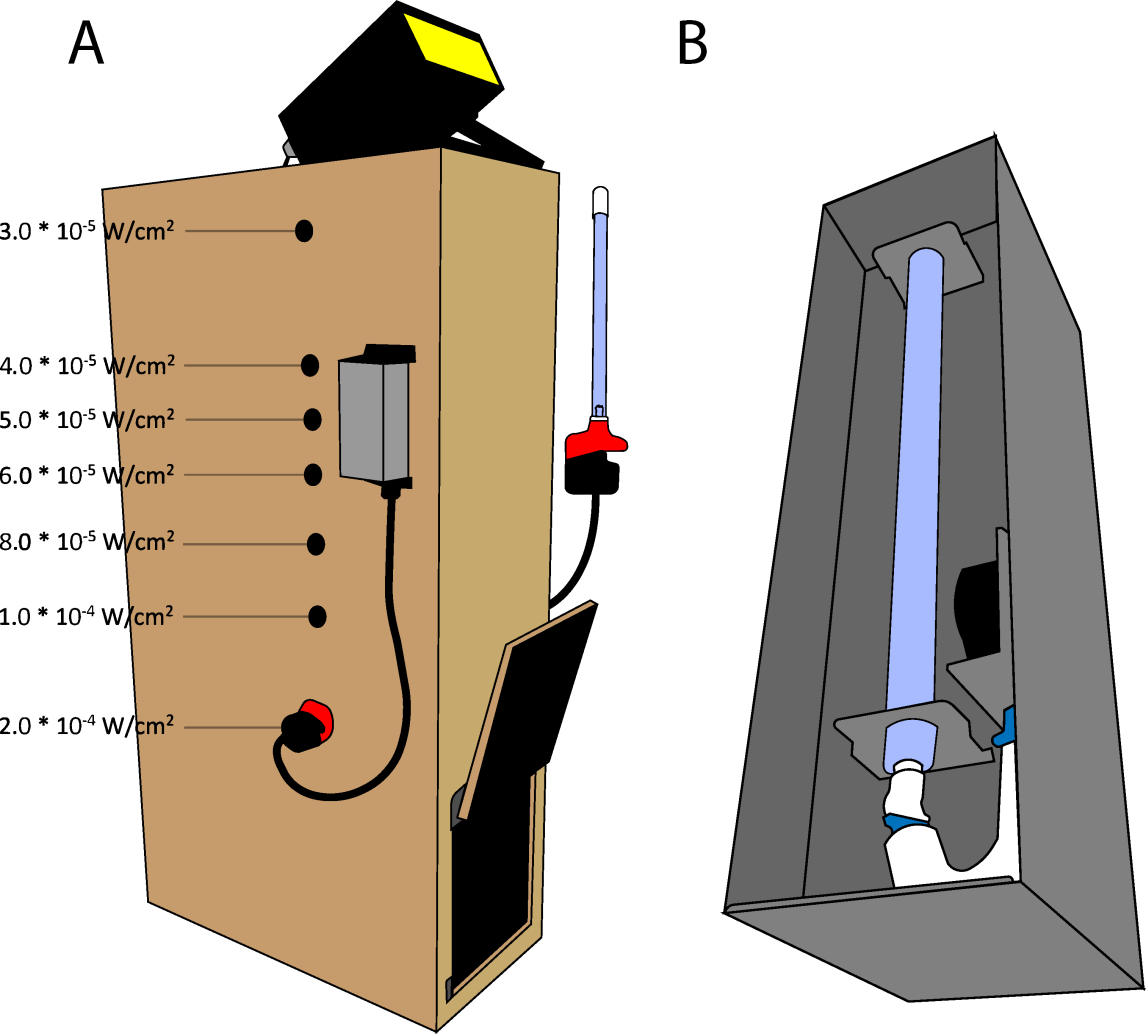
(**A**). Illustration of the chamber built for irradiation under 254 nm UV, which contains a locking door for sample loading and the power supply for a Viqua VH200 UV bulb, model 602805, illustrated both as loaded into the lowest chamber slot and free at right is mounted to the side. Seven holes corresponding to various irradiance values allow the UV bulb to mount horizontally through the chamber to irradiate the sample. The holes are sealed when not in use to eliminate ambient light penetration. An ILT-1700 radiometer is mounted to the top of the chamber to monitor the D.C. output value of the UV source. (**B**). Illustration of the SterilRay microbe buster ballast and bulb. The ballast was placed at appropriate distances from the samples using a modular shelf. The 222 nm and 282 nm bulbs are interchangeable and screw into mounts inside the ballast.

Oocysts were treated in opaque 96-well, flat-bottom optical plates. The opacity of the wells prevented UVR penetrance across rows, with each row receiving a unique treatment (distance from source * exposure time). 254 nm UVR was generated using a Viqua VH2000 UV bulb (model 602805) and administered using a custom-built plywood chamber (Fig. 5A). On opposing walls of the chamber, 1/2-inch diameter holes were drilled to position the bulb at various distances from the sample (range = approximately 45–110 cm), enabling delivery of a series of static irradiances (W cm^−2^) as measured by an International Light Technologies ILT1700 radiometer equipped with a SED240 detector in a dark room. The calibration factor for the detector was set to the corresponding wavelength for testing each of the three wavelengths: 222 nm, 254 nm, and 282 nm. Radiant energy density was calculated by multiplying the recorded irradiation by the exposure time in seconds and a range of doses (fluences; J cm^−2^) was obtained by exposing the oocysts for 60, 180, or 300 seconds at each distance. The turbidity of each suspension was measured by taking the OD_600_ using a BioTek Cytation 5 (Agilent) plate reader.

For 222 and 282 nm treatments, a Sterilray MicrobeBuster excimer bulb apparatus (Fig. 5B) was used, with the ballast housing positioned at various distances from the sample using a modular rack (range = approximately 41–94 cm). Following treatment, oocysts were mixed 1:1 with 5% potassium dichromate (final concentration 2.5%) or water and incubated in the dark at 25°C for 7 days. Sporulation was evaluated by imaging at 40× magnification using an Olympus FV1000 confocal microscope followed by manual scoring. The criterion for scoring an oocyst as sporulated was the identification of four distinct sporocysts within the oocyst wall. The total number of sporulated oocysts was divided by the total oocyst count per image field to obtain the proportion of sporulated oocysts per image. Three counts to ≥100 total oocysts were scored each for each dose. For example, if 47 sporulated oocysts were counted out of a total of 125 oocysts over six fields of view, the sporulation rate would be scored as 47/125 = 0.376, or 37.6%.

### Infectivity assay

Broiler chickens (2 weeks old, *n* = 3/treatment, HR308, Longeneckers Hatchery, Elizabethtown, PA) were inoculated orally with 10^2^ *E. acervulina* oocysts. Chickens received either treated oocysts, which were exposed to 222 nm, 254 nm, or 282 nm UV at fluences of 0.065 or 0.125 mW cm^−2^ for 300 seconds (19.5 or 37.5 mJ cm^−2^, respectively) untreated oocysts, or no oocysts (controls). Based on prior experience, an inoculation dose of 10^2^ *E. acervulina* oocysts allows for comparisons of oocyst fecundity but is not high enough to cause clinical signs such as intestinal lesions. All inoculation of chickens was conducted under the Beltsville Agricultural Research Center (BARC) Animal Care and Use Protocol No. 22-06. Fecal material was collected between days 5–7 post-inoculation and processed for *Eimeri*a oocysts, using standard procedures for enumeration on a McMaster chamber. Duplicate counts were performed for each sample, and the mean and standard deviation of oocyst output/chick were calculated for each group.

### Statistical analysis

Statistical analyses were performed using SPSS 29 (47). Statistical significance was identified at *P* < 0.05.

## Data Availability

All data will be made available upon reasonable request to the corresponding author.
